# Treating Rare Diseases in Africa: The Drugs Exist but the Need Is Unmet

**DOI:** 10.3389/fphar.2021.770640

**Published:** 2022-01-10

**Authors:** Lucio Luzzatto, Julie Makani

**Affiliations:** ^1^ Department of Haematology and Blood Transfusion, Muhimbili University of Health and Allied Sciences, Dar-es-Salaam, Tanzania; ^2^ University of Florence, Florence, Italy

**Keywords:** sickle cell disease, paroxysmal nocturnal haemoglobinuria, hydroxyurea, eculizumab, orphan drugs, public health *versus* profit, cost of production-price mismatch, post-colonial debt

## Abstract

Rare diseases (RD) pose serious challenges in terms of both diagnosis and treatment. Legislation was passed in the US (1983) and in EU (2000) aimed to reverse the previous neglect of RD, by providing incentives for development of “orphan drugs” (OD) for their management. Here we analyse the current situation in Africa with respect to (1) sickle cell disease (SCD), that qualifies as rare in the US and in EU, but is not at all rare in African countries (frequencies up to 1–2%); (2) paroxysmal nocturnal haemoglobinuria (PNH), that is ultra-rare in Africa as everywhere else (estimated <10 per million). SCD can be cured by bone marrow transplantation and recently by gene therapy, but very few African patients have access to these expensive procedures; on the other hand, the disease-ameliorating agent hydroxyurea is not expensive, but still the majority of patients in Africa are not receiving it. For PNH, currently most patients In high income countries are treated with a highly effective OD that costs about $400,000 per year per patient: this is not available in Africa. Thus, the impact of OD legislation has been practically nil in this continent. As members of the medical profession and of the human family, we must aim to remove barriers that are essentially financial: especially since countries with rich economies share a history of having exploited African countries. We call on the Global Fund to supply hydroxyurea for all SCD patients; and we call on companies who produce ODs to donate, for every patient who receives an expensive OD in a high income country, enough of the same drug, at a symbolic price, to treat one patient in Africa.

## Introduction

The challenges posed by Rare Diseases have evolved in recent times in at least three ways. 1) Improved diagnosis. When the older between us was a medical student, identifying a patient with, for instance, Fanconi anaemia or Fabry disease, was regarded as an achievement of clinical acumen supported by specialized laboratory methodology: the diagnosis was often made by the individual effort of an obsessed clinical investigator. Now, in many cases, a clinical suspicion triggers DNA testing of an appropriate gene panel that can quickly confirm or refute the suspicion. 2) Increased awareness and patient empowerment. There is now a vast number of formally constituted or informal Patient Groups: a healthy development in our view. ORPHANET (https://www.orpha.net/consor/cgi-bin/index.php?lng=EN), founded in France, is now a global organization, particularly active in EU, that lists over 7,000 rare diseases and provides a wealth of information and activities; NORD (https://rarediseases.org) has a similar role in the US. 3) Legislation in the US and in Europe has introduced “Orphan Drug Designation” by FDA and EMA: in essence, a set of financial and regulatory incentives for drugs invented or re-purposed for the treatment of rare diseases.

The definition of rare disease is based on epidemiology: *i.e.,* less than 200,000 patients overall in the US; less than five in 10,000 in EU. These are clearly arbitrary cut-offs, and they are population-sensitive. A paradigmatic example is sickle cell disease (SCD): it qualifies as rare disease in the North of the world, but it is not at all rare in parts of India and particularly in tropical Africa, where the incidence of sickle cell disease (SCD: including the types SS, SC and S-thalassaemia) is of the order of 1%, and in some countries up to 2% (Piel et al., 2010). A second example is paroxysmal nocturnal haemoglobinuria (PNH): a disease that is ultra-rare throughout the entire world (Luzzatto et al., 2011a); recently there have been impressive developments in the management of PNH.

The purpose of this paper is to outline, for these two rare diseases, the gaps between optimal current management and current reality in Africa; and to make practical proposals aiming to ameliorate the situation.

With respect to optimal management we have referred to a vast literature, with due attention to recent authoritative reviews for SCD (*e.g.* (Piel et al., 2017; Ware et al., 2017)) and PNH (*e.g.* (Hill et al., 2017; Patriquin et al., 2019)). We then analysed what actually happens in Africa: again based on the literature, drawing also from our personal experience. In Tanzania the burden of SCD is high, and a programme aiming to combine clinical care, research, training and advocacy has been running for over 15 years (Makani et al., 2018) and it has become within the continent a hub of wider cooperation named *SickleinAfrica* (Makani et al., 2020).

### Sickle Cell Disease

SCD claims more than one “first” in medicine. The very term molecular disease was coined when it was discovered that the basis of SCD was a structural abnormality in the haemoglobin molecule in red cells (Pauling et al., 1949) (Ingram, 1956); then one allele of a DNA restriction fragment length polymorphism was the first found to be genetically linked to the haemoglobin S *HBB* ^*E6V*^ mutation (Kan and Dozy, 1978): this eased the diagnosis of SCD at the DNA level, facilitating prenatal diagnosis (Kazazian et al., 1972); at the same time, it opened up the vast research field currently known as genome wide association studies (GWAS). Prevention based on prenatal diagnosis has been widely successful for thalassemia in Sardinia (Cao and Kan, 2013) and in Cyprus (Bozkurt, 2007); but only in Cuba (another island) for SCD (Marcheco-Teruel, 2019). This important topic falls outside the scope of this paper.

**FIGURE 1:**
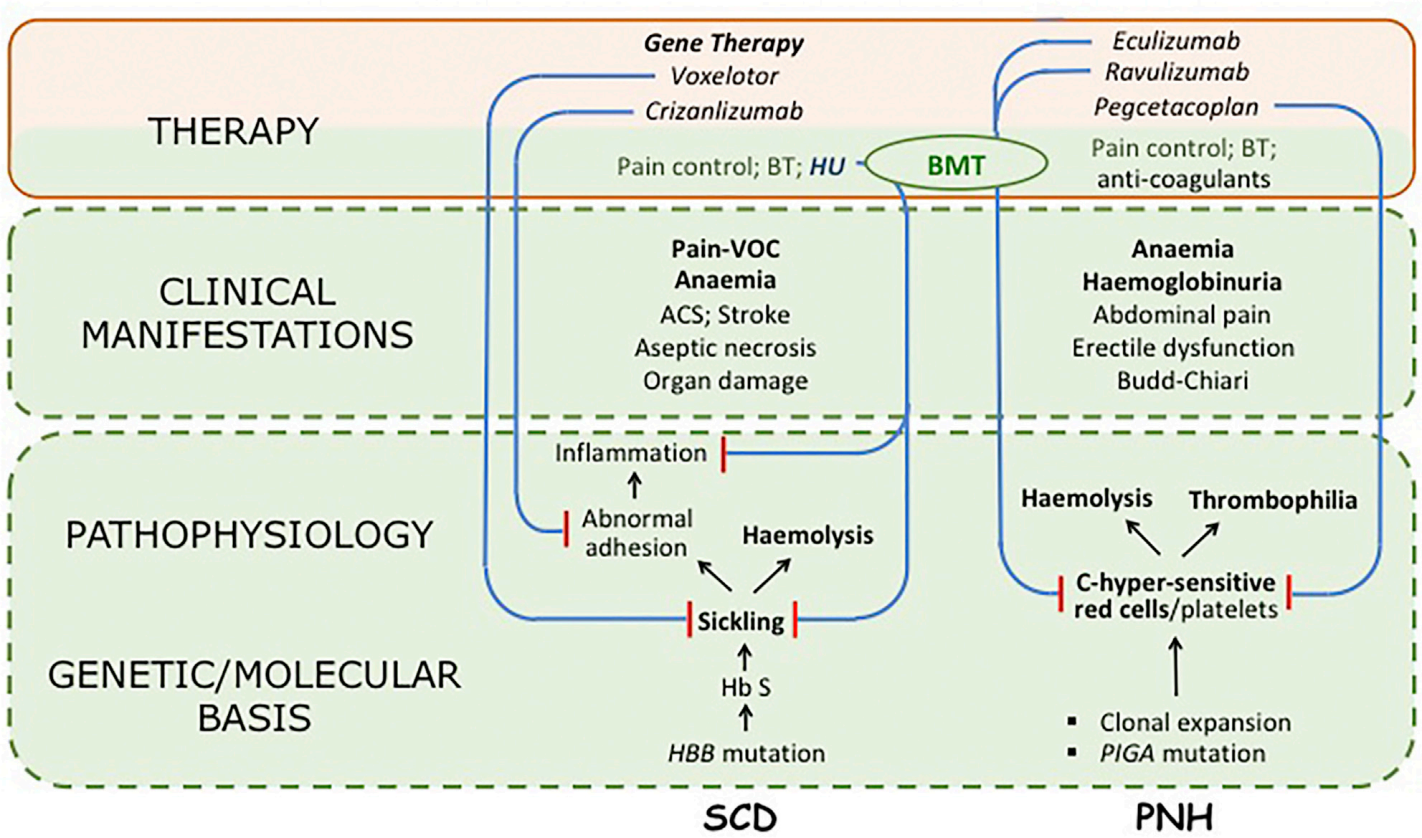
Synoptic view of two Rare Diseases, sickle cell disease (SCD) and paroxysmal nocturnal haemoglobinuria (PNH), and of drugs used in their treatment. For both diseases the *pathophysiology* is complex; but in SCD the primary problem is clearly sickling, whereas other features are important consequences, particularly a chronic inflammatory state; in PNH the primary abnormality is in the deficiency of the GPI-linked surface proteins CD55 and CD59, that make both red cells and platelets exquisitely sensitive to activated complement (C) (although it is not yet certain whether the marked thrombophilic tendency is a direct consequence of the platelet abnormality or an indirect consequence of intravascular haemolysis). For both diseases only major clinical manifestations are listed (VOC stands for vaso-occlusive crisis). Regarding therapy, long-established supportive measures are in green type; drugs are in italic; curative treatments are in bold: all other treatments are disease-modifying. Blue lines ending in red bars indicate the mechanism of action of targeted drugs; in PNH each drug inhibits in one way or another activation of complement (eculizumab and ravulizumab bind to C5; pegcetacoplan binds to C3: for details see (Luzzatto, 2021)). BT stands for blood transfusion; BMT indicates bone marrow transplantation (used successfully in both SCD and PNH). The area shaded in light green reflects the body of work that has led to identification of the molecular basis of the disease including the genetics, biochemistry and pathophysiology of SCD and PNH; there is also the body of experience regarding their clinical features, acquired over decades (or centuries) of basic and clinical research. The area shaded in light pink comprises innovative therapeutic measures produced by PHARMA, based on what was known in the green area, with the incentives and advantages afforded by Orphan Drug Designation (ODD). We are unable to provide, for either SCD or PNH, a$ figure for what is in green, and for what is in pink; however, we contend that a 5:1 ratio may not be an over-estimate.Hydroxyurea (HU) is an important case in point: this compound has been a drug for three-quarters of a century, and has been used in SCD over the last quarter. However, a new formulation, Siklos (containing in one capsule either 1,000 mg or 100 mg instead of 500 mg) has received designation as an orphan drug: the price per Gram is $16.50 instead of about $1. In practice, Siklos (not shown in the figure) has managed to move HU from the green area to the pink area.

With respect to management of SCD, for decades it has consisted only in the treatment of symptoms, of exacerbations, and of complications (Figure 1). Since the eighties bone marrow transplantation (BMT) was introduced as a curative approach (Vermylen et al., 1994); and a recent review has reported it can cure the disease in 90% of cases (Iqbal et al., 2021). However, for a variety of reasons only a small minority of patients receive BMT (Bolaños-Meade and Brodsky, 2014): including in the US, where the average cost of this procedure is in the range of $200,000–400,000. A survey of three sites in LMICs yields instead a cost of less than $15,000 (Faulkner et al., 2021). Based on this last figure, if a child were diagnosed with SCD at the age of 2, and if she/he were to take HU regularly (see below), by the age of 50 the expenditure on HU would be roughly the same as if BMT had been carried out at the time of diagnosis.

BMT services are already established in six African countries (Harif et al., 2020), but in only one, Nigeria (Bazuaye et al., 2014), of those with a high prevalence of SCD^1^. To date, 17 patients with SCD have received BMT, and 14 have been cured without serious complications^2^. Thus, curative treatment of SCD is achievable in Africa, and it is an example for other centres to follow. An outstanding issue is that patient selection ought to be based on clinical criteria rather than on the ability of an individual patient to pay.

Gene therapy for SCD (see Figure 1) (Ribeil et al., 2017; Abraham and Tisdale, 2021) is a hi-tech procedure whereby the patient’s haematopoietic stem cells are ‘mobilized’, then purified, transduced *in vitro* with an appropriate vector, checked for successful vector integration, and finally re-infused into the patient. From the clinical point of view this is in essence a modified auto-grafting procedure that requires myelo-suppression, but does not require a donor, does not require matching, and does not entail dire immune complications such as chronic graft versus host disease: therefore it may eventually surpass BMT. Gene therapy for SCD is marketed in the form of *Zynteglo*, at the price of $1.8 million per patient.

With respect to (non-curative) disease-modifying drugs, progress has been slow, until in 1995 the beneficial value of hydroxyurea^3^ (HU) was established (Charache et al., 1995). Over the past quarter-century HU has been used extensively in many countries: including *e.g.* Cuba, Central America (Svarch et al., 2006) and Brazil (Vicari et al., 2005), in children and in adults. There was no reason for not using it in Africa (Luzzatto et al., 2011b); but only recently highly significant beneficial effects have been formally demonstrated in a multi-centre trial involving Congo, Kenya, Uganda and Angola (Tshilolo et al., 2019). HU is effective as long as it is taken: that means, in most cases, for a lifetime. Since there are no published data on unselected patient populations, we have polled seven colleagues who have ample experience in three countries with high prevalence of SCD (Nigeria, DRC, Ghana). From the responses received and from our experience we estimate that SCD patients in Africa taking HU regularly are less than 20%.

The obvious question is: why? In a well-rehearsed analysis of potential ‘barriers’, the following have been listed (Adeyemo et al., 2019): lack of national guidelines, concerns about infertility, carcinogenic potential and side effects, high cost and unavailability of HU, difficulty in compounding paediatric dosages, need for toxicity surveillance, lack of time/skill to explain risks/benefits, insufficient experience or knowledge regarding mechanism of action, doubts about effectiveness, safety profile of HU in pregnancy and lactation, patients’ unwillingness. Clearly this is a mix of reservations of varying weight, some from patients and some from health workers: but buried in the midst is the stark item of cost. Quoting from another paper (Ryan et al., 2020): “Physicians only prescribed hydroxyurea therapy when they perceive the patient can afford the medicine; and patients reported they only use hydroxyurea therapy when they have funds to pay out-of-pocket”. In Africa the cost of 1 G of HU (the average daily dose for an adult with SCD) generally ranges from $0.5 to $1.0 (Costa et al., 2021). The problem of course is that, unlike with an acute disease, with SCD this cost must be born for a lifetime. The data above strongly suggest that in the vast majority of cases the main barrier that limits access to regular HU is financial. Indeed, when this medicine was produced locally by galenic compounding and offered to patients free of charge (Costa et al., 2021), the uptake was 100%. In Nigeria a local industry has been sensitive to the demand arising from a ‘rare disease’ that is not rare in that country, and has knocked down the price to $0.13 (see (Galadanci et al., 2019)): we can hope that others will follow this example.

### Paroxysmal Nocturnal Haemoglobinuria

PNH, like SCD, is a chronic haemolytic anemia; however, unlike SCD, it is acquired rather than inherited and, unlike SCD, it is an ultra-rare disease in every country of the world. For decades the only curative treatment has been BMT (Saso et al., 1999); the alternative was supportive treatment, including blood transfusion whenever necessary. PNH is a prototype of a non-malignant clonal disorder (Oni et al., 1970) that develops on a background of aplastic anaemia (Luzzatto and Risitano, 2018), through the expansion of a clone originating from a haematopoietic stem cell that has a somatic mutation in the *PIGA* gene (Takeda et al., 1993), that encodes an enzyme protein required for the biosynthesis of glycosyl-phosphatidyl-inositol (GPI). Since CD55 and CD59, two regulators of complement, are GPI-linked, they are deficient on the surface of PNH red cells: indeed hemolysis in PNH is complement-dependent. Peter Hillmen, in collaboration with Russell P Rother at Alexion, first explored complement blockade in PNH patients (Hillmen et al., 2004). Thereafter, a phase III trial of the anti-C5 monoclonal antibody eculizumab (ECU) (Hillmen et al., 2006) led to its approval by FDA and EMA as the first “PNH drug”,

The term revolutionary is sometimes over-used nowadays, but in this case it is a fact that the life of many PNH patients has been gratifyingly changed in quality, and also in duration (Kelly et al., 2011). In PNH C5 blockade, by preventing formation of the Membrane Attack Complex in the distal complement pathway (see Figure 1), abrogates intravascular haemolysis, the most disturbing pathophysiologic feature, that may contribute to life-threatening thrombosis. ECU also exemplifies how a major therapeutic innovation can be achieved: understanding the complement cascade, elucidating the pathogenesis of PNH, inventing monoclonal antibodies has required several decades of research in academic institutions; then, within a few years, a small company powered by state of the art technology was able to produce ECU. Although this successful combination did not arise through a deliberate public-private partnership, this is what it was. We can only applaud, until we look at the price: ECU is sold at about

$400,000 per patient per year. There are today in the world a few thousands patients who have received ECU for at least 10 years: each one of them has cost to a National Health Service in the EU, or to an insurer in the US, at least $4 million;

 despite the fact that ECU has had the perks of an orphan drug, and the company producing it has not paid any royalties to those who discovered PNH, complement, monoclonal antibodies. It just feels like there is something wrong here.

ECU has been a trailblazer. Soon after it was introduced, it became clear that preventing the haemolysis of PNH red cells had a down side: the un-lysed red cells are now opsonized by the complement component C3d, and thus become prey to macrophages (Risitano et al., 2009). This iatrogenic extravascular hemolysis is less severe and has less pathological consequences than the intravascular hemolysis of untreated PNH patients; but it has been a stimulus to try and do better. Three new molecules that target the proximal complement pathway, upstream of C5, are now on track to become medicines for PNH, and more are in the pipeline. ECU has been a trailblazer, unfortunately, also with respect to price: in this respect new molecules are likely to converge on using ECU as a benchmark. Indeed, pegcetacoplan (Hillmen et al., 2021), that is already FDA-approved, has a price-tag of $458,000 per year.

Since PNH is ultra-rare, it is not surprising that there have been very few cases reported from Africa (Manuel, 1969; Oni et al., 1970; Rizk et al., 2002; Lumori and Muyanja, 2019); in Tanzania we have so far diagnosed four patients (Ally et al., 2019). At the moment, as far as we know, ECU is not available in Africa, or in most Asian countries (including India and China), or in most countries in Latin America, and even in some EU countries. One of us, on account of age, has some experience of managing PNH from the pre-ECU era: but in one severe case we have formally requested from the Alexion company this drug on a compassionate basis, since it is not licensed in Tanzania: the request was declined.

## Discussion

The provision of medicines operates in today’s world within a framework that has a built-in source of conflict. Drugs produced by the pharmaceutical industry (PHARMA), owned and run by private enterprise, are then purchased and used by the health services that, in Europe (EU and UK), are public; in the US the health system is largely private (it is often referred to as the health industry), but with a substantial public component (the Veterans Administration, Medicare and Medicaid); while a variety of systems are operating in the rest of the world. Thus, National Health Services funded by taxpayers’ money must contend with PHARMA, that is legally entitled to earn maximum profit^4^. In the US the PHARMA industry and a large part of the health industry, that are both for profit, are frequently pitched against each other on account of drug prices.

This conflict poses serious problems. Eliminating profit from PHARMA did not work in the former Soviet Union, where the industry failed; but currently PHARMA maximizes profits by leveraging the fact that health services have an institutional obligation to provide the best care to all: this situation is fraught with risk, because the health services may collapse. We think that, as in many societal issues, human intelligence ought to find a balance: although, at the moment, there seems to be no mechanism in place to do so. FDA in the US and EMA in EU are doing generally a good job in assessing safety and effectiveness, but they are excluded from price negotiations. Although it is claimed that prices take into account value for money, assessment of value is based on dubious and ethically questionable quality-adjusted life-years (QALY); and there has been no agreement on the $ figure for 1 QALY^5^. The stark reality is that, at the moment, the price of drugs is dictated entirely by willingness to pay.

We think at least three points deserve consideration. First, whereas drugs are patented as inventions, they could not have been invented without an enormous body of knowledge that pre-existed^6^: see the pink area *versus* the green area in Figure 1. Second, the Orphan Drugs Act has been a success because patients receive new drugs, and PHARMA have discovered that investment in rare diseases–formerly a non-starter–can become a coveted area for venture capitalists: however, an Act that has offered incentives and benefits for developing a new drug, is silent about the basis on which that drug will be eventually priced (Luzzatto et al., 2018). Third, a recurrent objection to controlling prices is that this will limit profits accruing to investors, and high profits are precisely what is fuelling innovative drug development. However, all of the five top PHARMA companies spend less for R&D than they do for marketing (up to 42% of revenue: see https://www.pharmacychecker.com/askpc/pharma-marketing-research-development/)^7^.

We are not qualified to resolve, even in theory, this mega-conflict. As regards Africa, we cannot ignore the historical debt on the shoulders of ex-colonial powers that have exploited this continent for one century or longer. This debt has never been recognized on the legal level, and rarely on the political level. However, in the area of health there have been “aid” programmes: for instance, since 2002 the Global Fund has disbursed, in the fight against HIV, *tuberculosis* and malaria, more than $45 billion, 74% of which went to sub- Saharan Africa (https://www.theglobalfund.org/en/overview/). On a much smaller scale, an example worthy of note is that of imatinib: this drug is made available to patients with chronic myeloid leukaemia (CML) in Tanzania and in other countries through the glivec International’s Patient Assistance Program, established by Novartis and implemented in partnership with the Max Foundation (see (Nasser et al., 2021)). A limitation of these approaches is that the choices are made by the “donors”, not by the receivers: as a result we have to say–even though it sounds crude–that for a person in Tanzania it is financially preferable to have HIV disease or CML rather than SCD.

We think that one needs short-term devices and long-term solutions. In the short term, we call for SCD to be added to the agenda of the Global Fund. They have focused hitherto on three communicable diseases, based on the notion that they can be potentially eliminated more easily than an inherited disease: however, the reality in Africa is that we are very far from the elimination end-point, but at least the burden imposed on the population by these three diseases is being alleviated: exactly the same would be true for SCD if HU and other drugs were provided; and a good way to do this would be to give grants to local industry to produce them. At the same time, we call for a voluntary move by PHARMA, whereby for every patient with a rare disease who receives an expensive drug covered by NHS or by private insurance, the same drug should be provided at a symbolic price to one patient in a LMIC, particularly in Africa.

In the long term, we have no doubt that in Africa, like everywhere else, it is for each country’s government to look after the health of their people as a high priority–whether through a national health service or otherwise. In this respect, they will find ways to increase local production of medicines. With respect to expensive drugs for rare diseases, African countries, like the others, will have to decide how to negotiate prices with PHARMA: perhaps they will choose to do it through the African Union organization, that will be thus enabled to negotiate on behalf of 1.3 billion people.

## Data Availability

The original contributions presented in the study are included in the article/Supplementary Material, further inquiries can be directed to the corresponding author.

## Footnotes

1 At a conservative estimate the number of patients with SCD in Nigeria is 2.5 million: BMT has been received by probably less than 100.

2 This has been possible thanks to the indefatigable efforts of Nosa Bazuaye and his team at the University of Benin Teaching Hospital. For logistic reasons the procedures are currently carried out at the private Celltek Healthcare Medical Center, at a cost to each patient of $ 20,000, *i.e.* much less than if the patient had travelled in order to have BMT abroad.

3 HU is an inhibitor of ribonucleoside diphosphate reductase, and therefore of DNA synthesis. It has been used in the management of many malignant disorders, and today it is still standard of care in many patients with myeloproliferative neoplasms. One characteristic advantage of HU, compared to other chemotherapeutic agents, is that its toxicity is reversible. HU ameliorates SCD through at least two different mechanisms (see Figure 1). On one hand, by inhibiting cell division in the later stages of erythropoiesis, it favours the synthesis of foetal haemoglobin (Hb F): insertion of a Hb F molecule hinders polymerization of deoxy-Hb S, thus decreasing sickling (Faulkner et al., 2021). On the other hand, it decreases the neutrophil count, thus reducing the detrimental effects of the chronic inflammatory state that tends to be permanent in SCD patients (Harif et al., 2020).

4 In informal discussions we have heard PHARMA executives saying that if they relent on extracting the highest possible drug prices from “customers”, they might be sued for damages by shareholders.

5 The Incremental Cost-Effectiveness Ratio (ICER), an elaboration on QALY adopted by the National Institute for Health and Care Excellence (NICE), may be appropriate when one course of a drug can produce definitive cure (e.g. sofosbuvir for hepatitis C), or may prolong survival substantially (e.g. bevacizumab in cases of colon cancer); it breaks down for drugs that must be used for an indefinite period of time. Based on ICER, eculizumab at $400,000 per year should never have been approved for funding by the NHS.

6 The so-called R&D costs incurred to bring a drug to the market are in large part those of clinical trials. The average cost of a phase 3 trial has been estimated to be $19 million (Moore et al., 2018). Thus, it is a small component of the total cost of getting a drug approved by FDA, estimated to be 1−2 billion, and that includes, for instance, the compensation of the CEO of the company, that may be several million per year.

7 It is hard to understand why, in the case of rare diseases, any marketing is required at all. Expensive drugs for rare disease should be prescribed and managed by highly specialized professionals who are thoroughly familiar with new drugs within their specialty: either they have learnt current guidelines and recommendations, or they have written them.
